# Sputum RNA signature in allergic asthmatics following allergen bronchoprovocation test

**DOI:** 10.3402/ecrj.v3.31324

**Published:** 2016-07-13

**Authors:** Rob G.J.A. Zuiker, Catherine Tribouley, Zuzana Diamant, J. Diderik Boot, Adam F. Cohen, K. Van Dyck, I. De Lepeleire, Veronica M. Rivas, Vladislav A. Malkov, Jacobus Burggraaf, Marcella K. Ruddy

**Affiliations:** 1 Centre for Human Drug Research, Leiden, The Netherlands; 2 Merck Research Laboratories, Rahway, New Jersey, USA; 3 Novartis, New York, NY, USA; 4 Department of Respiratory Medicine and Allergology, Skane University Hospital, Lund, Sweden; 5 Department of Clinical & Pharmacology, University Medical Center Groningen, Groningen, The Netherlands; 6 Department of General Practice, University Medical Center Groningen, Groningen, The Netherlands; 7 QPS Netherlands, Groningen, The Netherlands; 8 Janssen Biologics B.V., Leiden, The Netherlands; 9 EMD Serono, Rockland, MA, USA

**Keywords:** allergen bronchial provocation test, asthma, sputum, Th2 inflammation, DNA microarray, fluticasone

## Abstract

**Background:**

Inhaled allergen challenge is a validated disease model of allergic asthma offering useful pharmacodynamic assessment of pharmacotherapeutic effects in a limited number of subjects.

**Objectives:**

To evaluate whether an RNA signature can be identified from induced sputum following an inhaled allergen challenge, whether a RNA signature could be modulated by limited doses of inhaled fluticasone, and whether these gene expression profiles would correlate with the clinical endpoints measured in this study.

**Methods:**

Thirteen non-smoking, allergic subjects with mild-to-moderate asthma participated in a randomised, placebo-controlled, 2-period cross-over study following a single-blind placebo run-in period. Each period consisted of three consecutive days, separated by a wash-out period of at least 3 weeks. Subjects randomly received inhaled fluticasone ((FP) MDI; 500 mcg BID×5 doses in total) or placebo. On day 2, house dust mite extract was inhaled and airway response was measured by FEV1 at predefined time points until 7 h post-allergen. Sputum was induced by NaCl 4.5%, processed and analysed at 24 h pre-allergen and 7 and 24 h post-allergen. RNA was isolated from eligible sputum cell pellets (<80% squamous of 500 cells), amplified according to NuGEN technology, and profiled on Affymetrix arrays. Gene expression changes from baseline and fluticasone treatment effects were evaluated using a mixed effects ANCOVA model at 7 and at 24 h post-allergen challenge.

**Results:**

Inhaled allergen-induced statistically significant gene expression changes in sputum, which were effectively blunted by fluticasone (adjusted *p*<0.025). Forty-seven RNA signatures were selected from these responses for correlation analyses and further validation. This included Th2 mRNA levels for cytokines, chemokines, high-affinity IgE receptor FCER1A, histamine receptor HRH4, and enzymes and receptors in the arachidonic pathway. Individual messengers from the 47 RNA signatures correlated significantly with lung function and sputum eosinophil counts.

**Conclusion:**

Our RNA extraction and profiling protocols allowed reproducible assessments of inflammatory signatures in sputum including quantification of drug effects on this response in allergic asthmatics. This approach offers novel possibilities for the development of pharmacodynamic (PD) biomarkers in asthma.

Inhaled allergen challenge can be applied to study the pathophysiology and the immune biology to allergic stimuli within the airways. Allergen challenge is highly reproducible and serves as an integral disease model enabling the investigation of several features of asthma (1). In drug development, allergen challenge is an established tool predicting clinical efficacy of novel anti-allergic and anti-asthma treatments (2). Hypertonic saline-induced sputum (3) has been shown to yield reproducible increases in inflammatory cells and biomarkers following allergen-induced late asthmatic response (LAR) (4) with subsequent response to novel and existing anti-inflammatory therapies (2, 4–7).

Microarray technology allows to profile gene expression of the entire genome and has been widely applied in several asthma studies (8, 9). A large majority of these gene profiling studies involved tissue obtained from asthmatics like airway epithelium (10, 11), bronchial biopsies (12), or nasal mucosal cells (13). Although gene expression has also been studied in fluids from asthmatics like blood (14), broncho-alveolar lavage (15, 16), and induced sputum (17), little is published on extensive gene expression profiling on induced sputum cells following allergen challenge.

In this study, Affymetrix 2.0 microarray technology was used to measure the gene expression levels of >50,000 transcripts in induced sputum obtained from 13 allergic asthmatics before and after allergen challenge. In a refined set of 47 genes signatures, we aimed to study: 1) the feasibility and reproducibility of quantification of gene expression in induced sputum at 7 and 24 h post-challenge, 2) their reversibility after a short course of inhaled fluticasone (FP) treatment, and 3) the correlation with lung function and eosinophil measurements.

## Methods

### Study population and design

**Fig. 1:**
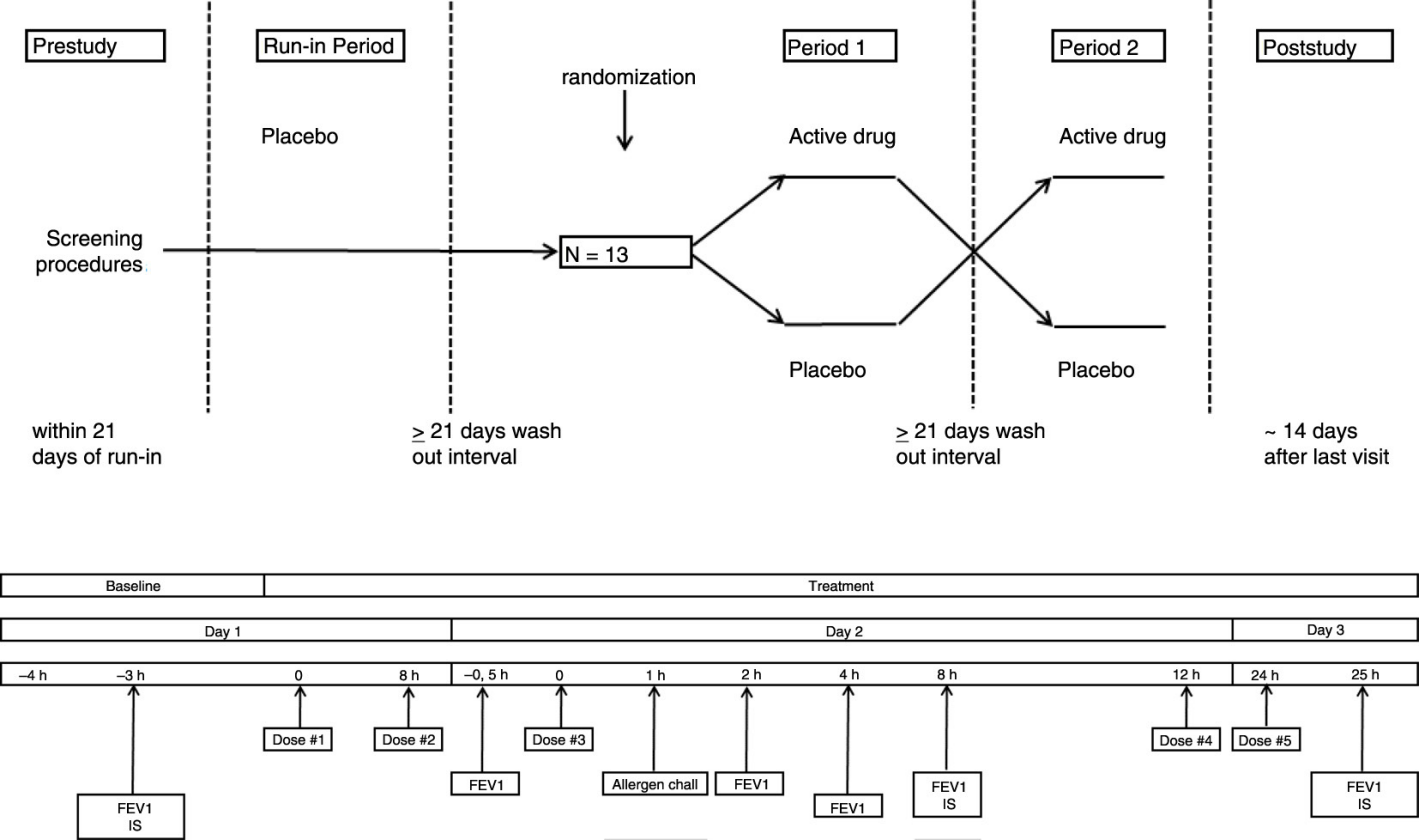
Study design. Overview of the single-blind placebo run-in period and double-blind cross-over study periods 1 and 2 (upper section). Overview of study assessments (lower section). Time zero is time of first study medication dosing. The single-blind placebo run-in screening period and the subsequent study periods 1 and 2 were identical. IS: induced sputum.

Thirteen non-smoking subjects with clinically stable, mild-to-moderate allergic asthma (18) using only *prn* short-acting beta2-agonists and with dual airway responses to inhaled house dust mite (HDM), documented during the single-blind placebo run-in screening period, participated in a double-blind, 2-way cross-over study. Each period consisted of three consecutive days, with ≥3 weeks wash-out between periods (Fig. 1). The screening was identical to the subsequent treatment periods during which subjects randomly received inhaled FP metered dose inhaler (MDI; 500 µg BID, total of 5 doses) or matching placebo. On day 1, baseline measurements, including spirometry and subsequent sputum induction (3×5 min NaCl 4.5%), were performed prior to study medication. On day 2, 1 h post-study medication, subjects underwent a titrated allergen challenge (1). The subsequent airway response was repeatedly measured by FEV1 until 7 h post-allergen. At 24 h post-allergen (day 3), test procedures were repeated as on day 1. All test procedures were conducted according to standardised, validated methods and at the same time of the day (within 2 h) during the different treatment periods (1, 19–21).

A dual airway response to inhaled HDM extract consisted of an early asthmatic response (EAR) and a LAR, defined as a fall in FEV1 ≥15% from baseline occurring between 0–3 h and 3–7 h post-allergen, respectively.

This study was part of an allergen study measuring allergen-induced Th2-profile in sputum. Detailed information from the same study on subject characteristics, reproducible quantification of sputum cytokines and chemokines, related allergen-induced airway responses, and their reversal by fluticasone, have been described in a recent publication (7).

The study was approved by the Ethics Committee of Leiden University Medical Center, Leiden, The Netherlands, and all participants gave a signed informed consent (EUDRACT number 2007-003671-40).

### Study medication

Fluticasone 250 µg/puff (Allen & Hanburys, Glaxo Wellcome Ltd, Middlesex, UK) and matching placebo (Armstrong Pharmaceuticals Inc., Canton, MA, USA, packaged at Merck Frosst, Kirkland, Canada) were supplied in identical MDIs and inhaled per single puff through an Aerochamber (Volumatic, GlaxoSmithKline, Zeist, The Netherlands).

### Allergen challenge

The allergen challenge was performed using the 2 min tidal breathing method that has been previously validated (1). The run-in period served as a dose (range) finding procedure, while during study periods 1 and 2, each subject inhaled the same 2 or 3 cumulative doses of the allergen extract that had caused a fall in FEV1 of at least 15% from baseline during the run-in period. Following diluent, incremental doubling concentrations (7.81–2,000 BU/mL) of HDM extract (*Dermatophagoides pteronyssinus*; SQ 503, ALK-BPT, ALK-Abelló, Almere, The Netherlands) in phosphate-buffered saline (PBS) were aerosolised by a calibrated jet-nebuliser (DeVilbiss 646, output 0.13 mL/min, Somerset, Pennsylvania, USA) and inhaled at approximately 12 min intervals, until the EAR was reached (defined as a decrease in FEV1 of ≥15% from post-diluent baseline within 1 h post-allergen). Airway response to inhaled allergen was measured by FEV1 in duplicate on a calibrated spirometer (Vmax Spectra; Sensor Medics, Bilthoven, The Netherlands) according to standard procedures (22), at 10, 20, 30, 45, 60, 90 and 120 min and then hourly until 7 h after the last allergen inhalation. The highest, technically valid measurement was expressed as percentage decrease from post-diluent baseline FEV1 and included into the analysis.

### Sputum induction, processing and analysis

Sputum induction was performed as previously described (21, 23) using a DeVilbiss Ultraneb 2,000 ultrasonic nebuliser (Tefa Portanje, Woerden, The Netherlands) connected to a 100-cm-long plastic tube, with an internal diameter of approximately 22 mm, connected to a two-way valve (No.2700; Hans-Rudolf, Kansas City, MO, USA) with a mouthpiece. Hypertonic saline (NaCl 4.5%) was nebulised and inhaled through the mouth, with the nose clipped, during three periods of 5 min. At approximately 7 min following each induction, spirometry was performed as a safety measure.

The cell pellet was processed as a full sample according to guidelines (21, 24). The processing took place within 2 h of collection. A DTT 0.1% solution (dithiothreitol, Calbiochem, La Jolla, CA, USA) was mixed with a protease inhibitor pill (Complete Protease Inhibitor Cocktail tablets, Roche Applied Science #11 697 498 001; 1 pill per 50 mL of solution). The volume of the entire sputum sample was determined and an equal amount of 0.1% DTT/protease inhibitor solution was added. Subsequently, the sample was mixed with a pipette and placed in a warm shaking bath for 15 min at 37°C. The homogenised mixture was centrifuged at 390G (1,500 rpm) during 10 min. The supernatant was removed.

To determine cell viability and the total cell count, the cell pellet was re-suspended in 2 mL PBS and filtered; 50 µL of the suspension was mixed with 50 µL of Trypan Blue. Total cell counts were determined in a counting chamber (Bürker; Omnilab 402521) using a cell counter (Omnilab 7005333). Cytospin slides (50 µL/cytospin; Shandon Cytospin 4, Thermon Electron Corporation, Runcorn, UK) were prepared by diluting the cell suspension with PBS in order to obtain approximately 0.5×10^6^ cells per mL, and subsequently centrifuged for 3 min at 254 G. Differential cell counts of eosinophils, neutrophils, lymphocytes, macrophages, epithelial and squamous cells were performed on May-Grünwald-Giemsa-stained cytospins by a certified cytopathologist. In each sputum sample, at least 500 nucleated cells, excluding squamous cells, were counted twice and the average percentage of each cell type was determined and expressed as percentage of nonsquamous cells. If >80% of the cell count consisted of squamous cells, the quality of the sputum sample was judged unsatisfactory and was excluded from analysis.

The remaining suspension was centrifuged a second time. The resulting cell pellet was re-suspended in 1 mL of TRIzol^®^ (Invitrogen, Cat. # 15596-018, Life Technologies, Carlsbad, California, USA). RNA was amplified using WT-Ovation^®^ amplification technology (NuGEN, San Carlos, California, USA). The amplified material was labelled and hybridised using a standard Affymetrix protocol. Gene expression studies were performed using the Rosetta/Merck Human RSTA Custom Affymetrix 2.0 microarray (Affymetrix, Santa Clara, California, USA) containing 51,562 probe sets interrogating 50,159 human transcripts predominantly from REFSEQ, GenBank, dbEST and ENSEMBL databases as described on the Gene Expression Omnibus website (www.ncbi.nlm.nih.gov/geo/). The accuracy of sample processing was monitored through quality metrics assessing RNA yield, RNA quality: 18S/28S ribosomal RNA ratio, RNA integrity number (RIN) score, and hybridisation parameters: 3′/5′ ratios for GAPDH mRNA and scale factor. In addition, the amount of bacterial RNA contamination was evaluated by calculating the area under the curve for the 16S and 23S (bacterial) versus the 18S and 28S (eukaryotic) ribosomal RNA peaks using a bio analyser electropherograms (Agilent, Santa Clara, California, USA). Specimens with more than 80% bacterial contamination were removed from the analysis. Data were normalised using the robust multichip average (RMA) algorithm prior to statistical analysis.

### Statistical model for data analysis

A mixed effect ANCOVA model was selected including terms for baseline gene expression, treatment, sequence and period as fixed effects and subject nested in sequence as a random effect. Gene expression change from the appropriate baseline was used as the dependent variable. The baselines for each of the periods were used as covariates.

### Analysis of treatment effects

For each time point, 7 and 24 h, the allergen challenge effect (ACE) and the FP treatment effect (FTE) were calculated. The ACE was calculated as the change from baseline when the subject received placebo treatment. The FTE was calculated as the difference in change from baseline between the FP treatment group and the placebo group. *p* Values for each gene in each treatment effect were adjusted using the Benjamini–Hochberg's procedure with a false discovery rate (FDR) level pre-specified at 0.025 to select significant genes.

### Correlation analyses

Pearson correlation coefficient and the associated *p* value were computed for correlation between the estimated individual subject-level effect, separately for ACE and FTE, for a given clinical endpoint and gene of interest. Assuming no period or sequence effect, subject-level ACE was calculated as the log-transformed change from baseline, for a clinical endpoint or gene of interest, when the subject received placebo treatment. Similarly, subject-level FTE was calculated as the difference in change from baseline for a clinical endpoint or gene of interest when the subject received fluticasone versus placebo. Type I error of 10% (two-sided) was used to select significant results, and no multiplicity adjustment was applied for declaring statistical significance.

## Results

Inhaled HDM induced both an EAR and an LAR in all subjects during both placebo periods. Compared to placebo, fluticasone significantly (*p*<0.001) reduced the EAR and completely blunted the LAR (7).

Sputum samples were analysed from asthmatic subjects who provided a baseline specimen in period 1 and period 2, and that passed the quality control (Table 1). Out of 13 subjects, nine had evaluable samples at both baselines. An insufficient amount of human mRNA was the main cause of sample exclusion, along with samples that did not meet the criteria for matrix microarray quality control.

**Table 1:** Treatment allocation and sample quality results

Subject	Run-in, 0 h	Run-in, 7 h	Run-in, 24 h	Period 1, 0 h	Period 1, 7 h	Period 1, 24 h	Period 2, 0 h	Period 2, 7 h	Period 2, 24 h
1	Baseline	Placebo	Placebo	Baseline	Placebo	Placebo	Baseline	Fluticasone	Fluticasone
2	Baseline	**Fail**	Placebo	**Fail**	Fluticasone	**Fail**	Baseline	**Fail**	Placebo
3	Baseline	Placebo	Placebo	Baseline	Fluticasone	Fluticasone	Baseline	Placebo	Placebo
4	Baseline	Placebo	Placebo	Baseline	Placebo	Placebo	Baseline	Fluticasone	Fluticasone
5	Baseline	Placebo	Placebo	Baseline	Placebo	Placebo	Baseline	Fluticasone	Fluticasone
6	Baseline	Placebo	Placebo	Baseline	Fluticasone	Fluticasone	Baseline	**Fail**	Placebo
7	**Fail**	**Fail**	**Fail**	**Fail**	**Fail**	**Fail**	**Fail**	**Fail**	**Fail**
8	Baseline	Placebo	Placebo	**Fail**	**Fail**	Fluticasone	Baseline	Placebo	Placebo
9	**Fail**	**Fail**	**Fail**	**Fail**	**Fail**	**Fail**	**Fail**	**Fail**	**Fail**
10	Baseline	Placebo	Placebo	Baseline	Placebo	Placebo	Baseline	Fluticasone	Fluticasone
11	Baseline	Placebo	Placebo	Baseline	Fluticasone	Fluticasone	Baseline	Placebo	Placebo
12	Baseline	Placebo	**Fail**	Baseline	**Fail**	Placebo	Baseline	Fluticasone	**Fail**
13	Baseline	Placebo	Placebo	Baseline	Fluticasone	Fluticasone	Baseline	Placebo	Placebo

Fail: the majority of failures was due to insufficient amount of human mRNA; few failures were because samples did not pass the matrix microarray quality control.

**Fig. 2:**
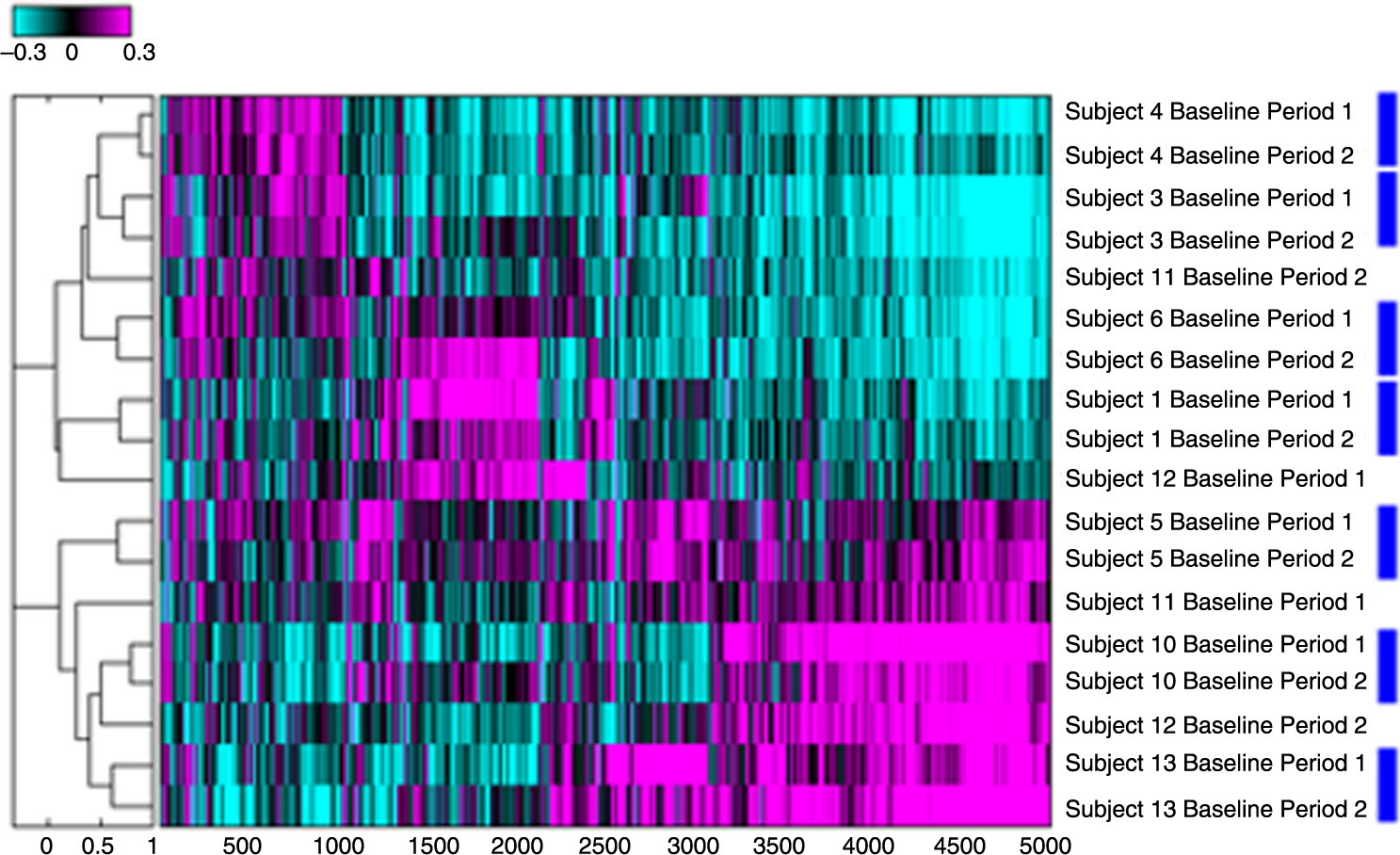
Hierarchal cluster assessment for reproducibility of sputum microarray data. Analysis included only subjects for which the baseline specimens at period 1 and 2 were available. Numbers refer to subject numbers. Log 10 ratios of intensity estimates versus the average of all intensities are displayed. Magenta colour refers to probe sets that are up-regulated in reference to the pool of all specimens analysed and cyan to the probe sets that are down-regulated. Blue rectangles link specimens from the same subject that co-cluster on the dendrogram.

The reproducibility of the sputum induction and collection procedures for RNA profiling after hybridisation on microarrays were evaluated by comparing individual gene expression intensities in combination with hierarchical clustering using Pearson correlation coefficients (Fig. 2) (25). The results of this cluster analysis revealed that 14 out of 18 sputum specimens clustered appropriately in subject specific pairs, validating our sputum collection and isolation protocol.

**Fig. 3:**
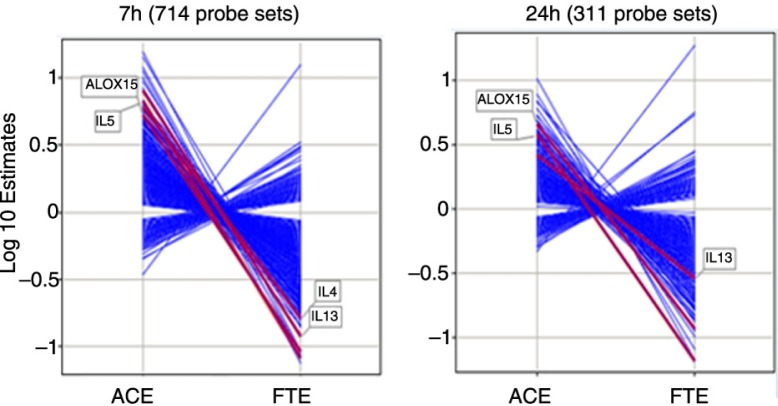
Log 10 estimates of gene expression changes for the significant genes identified from contrast analysis at 7 hours and 24 hours with an FDR < 0.025. ACE: allergen challenge effect, estimates of changes from baseline in the placebo group. FTE: fluticasone effect, estimates of differences in change from baseline between the placebo and the fluticasone groups.

The whole microarray contained 51,562 probe sets. At 7 h post-allergen challenge, and applying an FDR of < 0.025, a total of 4.175 and 1.001 statistical significant probe sets were identified for the allergen effect (ACE) and the FTE, respectively. Likewise, 1.143 and 1.018 statistical probe sets were identified at 24 h post-allergen for the allergen effect and the FTE, respectively. Seven hundred fourteen probes sets were regulated by both the ACE and FP treatment at 7 h and 311 probe set at 24 h post-challenge. All of the genes regulated by both the allergen challenge and FP at each time point were reversed from their allergen-induced levels in presence of fluticasone (Fig. 3). In other words, fluticasone effectively blunted the response to the allergen challenge at the gene expression level.

**Fig. 4:**
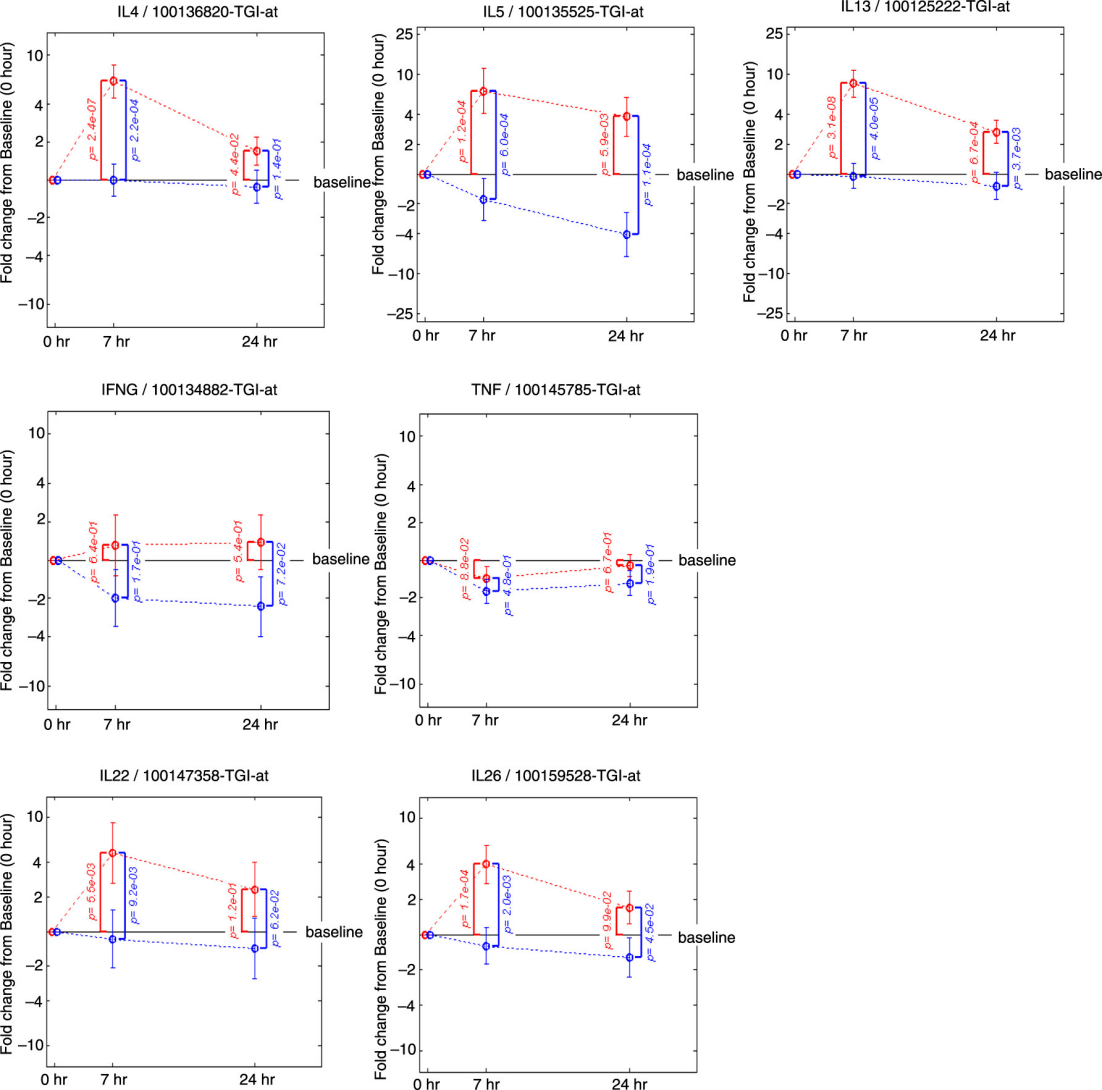
**Fold change from baseline in gene expression**. Th2 mRNA levels (IL4, IL5, IL13), Th1 mRNA levels (IFNG and TNF), Th17 mRNA levels (IL22, IL26). Fold change from baseline for the placebo group is represented in red. Fold change from baseline for the fluticasone group is represented in blue. *p* Values are adjusted *p* values; error bars represent 90% confidence intervals.

**Fig. 5:**
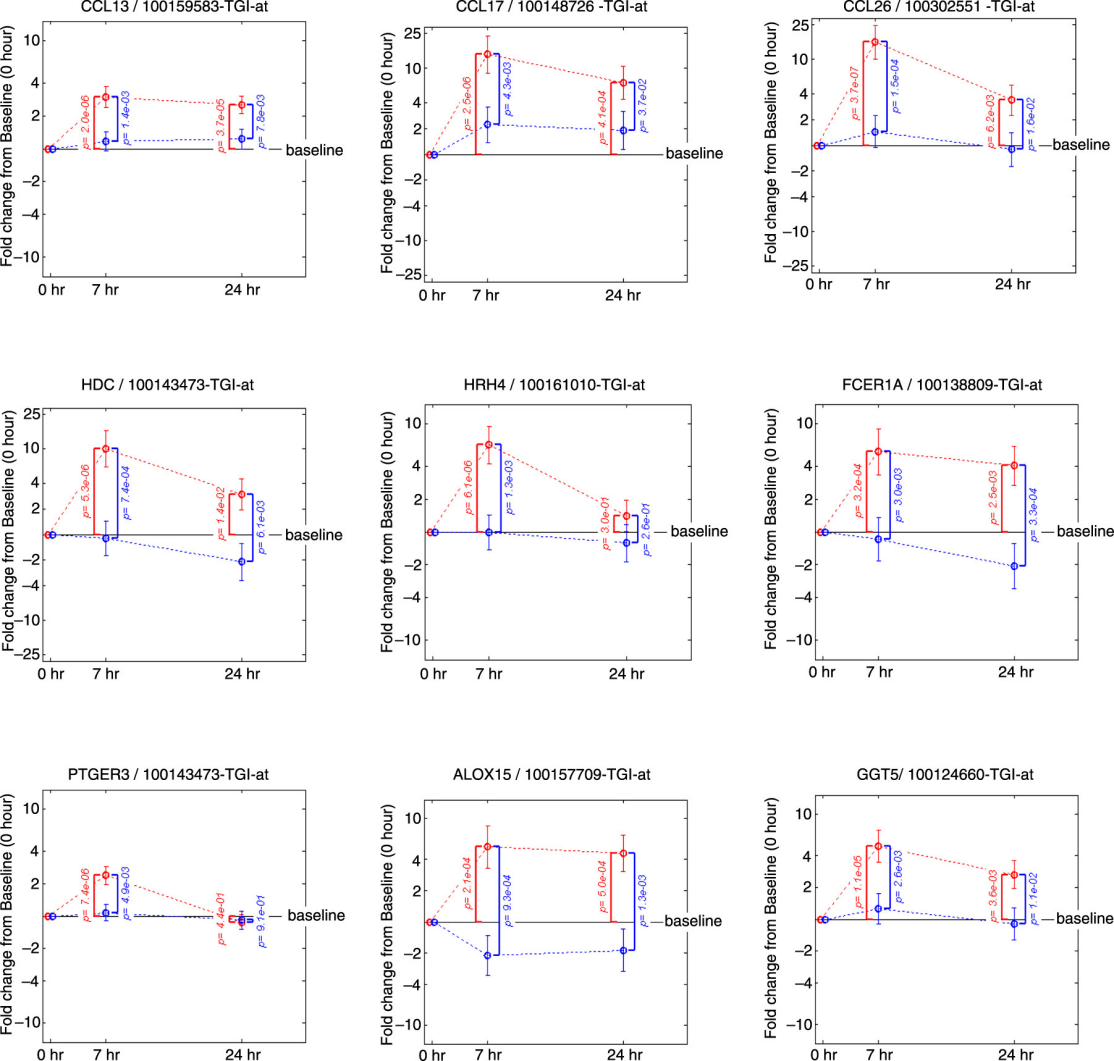
**Fold change from baseline in gene expression**. Inflammatory chemokines (CCL13, CCL17, CCL26), molecules controlling the release of histamine (HDC, HRH4, FCER1A) prostaglandins and leukotrienes (PTGER3, ALOX15, GGT5). Fold change from baseline for the placebo group is represented in red. Fold change from baseline for the fluticasone group is represented in blue. *p* Values are adjusted *p* values; error bars represent 90% confidence intervals.

Quantification of the individual genes that contribute to the key mRNA levels for the Th1, Th2 and Th17 pathways was performed by displaying the change from baseline in gene expression at 7 and 24 h following allergen challenge in presence or absence of FP treatment (Fig. 4). This analysis revealed the up-regulation by the allergen challenge and the down-regulation by FP treatment of the gene expression for several key Th2 mRNA levels for interleukin (IL)-4, IL-5 and IL-13 and an absence of an effect on key Th1 mRNA levels for interferon (INF)-γ and tumour necrosis factor (TNF). Chemokine ligand 13 (CCL13)/monocyte chemoattractant protein (MCP)-4 (26), CCL17/thymus and activation regulated chemokine (TARC) (27) and CCL26/eotoxin-3 (28) are inflammatory chemokines mediating Th2 cell recruitment and known to be induced by IL-4. Their gene expressions were up-regulated by the allergen challenge and down-regulated by FP treatment following a similar pattern as the Th2 mRNA levels (Fig. 5). Likewise, the same pattern was observed for genes belonging to pathways controlling the release of inflammatory parameters like: HDC (histidine decarboxylase) known to catalyse the production of histamine (29); histamine receptor 4 HRH4 which is specific for eosinophils and basophils (30); FCER1A, the alpha subunit of the high-affinity IgE receptor which directly binds IgE and through crosslinking induces the release of preformed histamine and proteases as well as the generation of leukotrienes and prostaglandins; the messengers for the enzyme GGT5 (gamma-glutamyl transferase 5, which converts leukotrienes C4 to D4) (31); ALOX15 (15-lipoxygenase) and the receptor PTGER3 (prostaglandin receptor 3) were also up-regulated by the allergen challenge and down-regulated by FP treatment. In most of the cases, the fold change from baseline was higher at 7 h versus 24 h and the *p* values smaller. This suggests that the 7 h time point provides the most useful readouts of the strict inflammatory response following an allergen challenge.

**Fig. 6:**
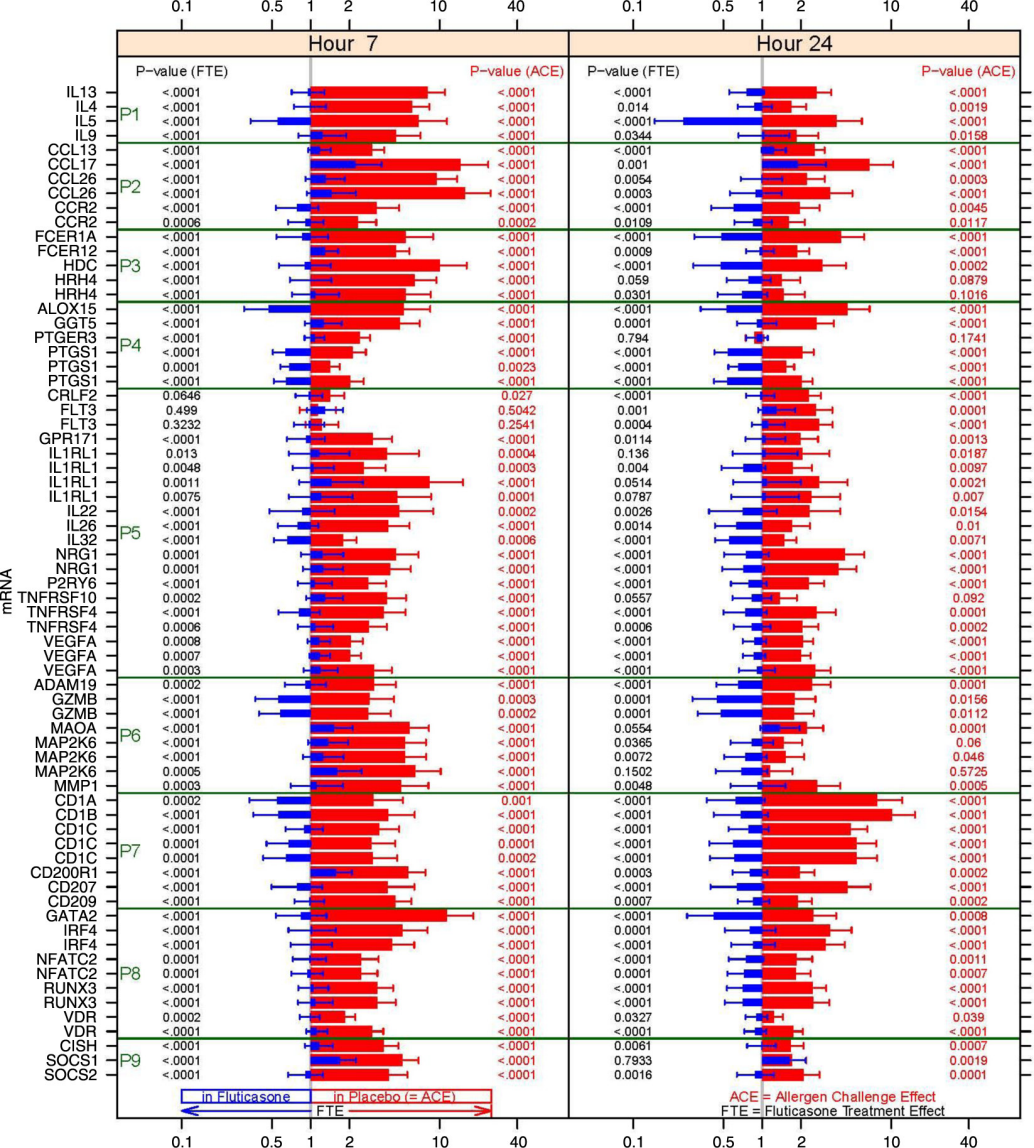
Fold changes over baseline (point estimate and 90% confidence intervals) for the 47 RNA signatures. Red bars represent the change from baseline in the placebo group and blue bars in the fluticasone group. *p* Values for the allergen challenge effect (ACE) and the fluticasone treatment effect (FTE) are represented in red on the right hand side and in black on the left hand side, respectively. P1: mRNA levels for Th2 cytokines; P2: chemokines and chemokine receptors; P3: FCERI and histamine signalling; P4: enzymes and signalling molecules in prostaglandin, leukotriene pathways; P5: other mRNA levels for cytokines, growth factors and their receptors; P6: other enzymes; P7: membrane-bound glycoproteins; P8: transcription factors; P9: regulators of the inflammatory response.

In order to facilitate the correlation analyses, the union of the genes affected by the allergen challenge and fluticasone 7 or 24 h post-challenge was reduced to a set of 47 RNA signatures based on statistical significance, intensity of the change from baseline, biological relevance and classified based on druggable structural and functional categories (Table 2 and Fig. 6). All the genes represented in the 47 RNA signatures harbour robust expression changes, and the large majority of them is up-regulated after 7 h with the exception of FLT3 and CRLF2, which are regulated only after 24 h.

**Table 2:** Change from baseline in the placebo and the prednisone group for the 47 RNA signatures

			Point estimate (90% confidence interval)		
			
Probe set	Gene symbol	Hour^a^	Fold change over baseline in placebo group (P)	Fold change over baseline in fluticasone group (F)	F/P
Th2 cytokines					
100125222_TGI_at	IL13	7	**8.08 (5.92, 11.01)**	0.95 (0.71, 1.28)	0.12 (0.08, 0.18)
		24	2.64 (2.03, 3.43)	0.76 (0.55, 1.03)	0.29 (0.19, 0.44)
100135525_TGI_at	IL5	7	**6.81 (4.08, 11.38)**	0.56 (0.34, 0.91)	0.08 (0.04, 0.16)
		24	3.77 (2.39, 5.93)	0.25 (0.15, 0.41)	0.07 (0.03, 0.12)
100136820_TGI_at	IL4	7	**6.1 (4.48, 8.3)**	0.99 (0.74, 1.32)	0.16 (0.1, 0.25)
		24	1.69 (1.3, 2.19)	0.88 (0.64, 1.19)	0.52 (0.34, 0.79)
100142120_TGI_at	IL9	7	**4.57 (2.94, 7.11)**	1.23 (0.8, 1.88)	0.27 (0.17, 0.43)
		24	1.84 (1.24, 2.73)	1.03 (0.66, 1.62)	0.56 (0.36, 0.87)
Chemokines and chemokine receptors					
100124067_TGI_at	CCR2	7	1.47 (1.22, 1.77)	1.09 (0.91, 1.3)	0.74 (0.59, 0.93)
		24	1.24 (1.04, 1.47)	1.02 (0.84, 1.23)	0.82 (0.65, 1.03)
100142511_TGI_at	CCL26	7	**9.41 (6.47, 13.68)**	1.3 (0.91, 1.85)	0.14 (0.09, 0.22)
		24	2.21 (1.6, 3.05)	0.99 (0.68, 1.43)	0.45 (0.29, 0.7)
100147484_TGI_at	CCR2	7	3.25 (2.19, 4.84)	0.79 (0.54, 1.15)	0.24 (0.16, 0.37)
		24	1.95 (1.36, 2.79)	0.6 (0.4, 0.9)	0.31 (0.2, 0.47)
100148726_TGI_at	CCL17	7	**14.44 (8.76, 23.79)**	2.21 (1.38, 3.56)	0.15 (0.08, 0.28)
		24	**6.75 (4.38, 10.4)**	1.89 (1.14, 3.12)	0.28 (0.16, 0.49)
100159583_TGI_at	CCL13	7	3 (2.42, 3.71)	1.17 (0.96, 1.44)	0.39 (0.3, 0.51)
		24	2.53 (2.1, 3.04)	1.23 (0.99, 1.52)	0.49 (0.37, 0.63)
100302551_TGI_at	CCL26	7	**15.7 (9.93, 24.84)**	1.45 (0.93, 2.24)	0.09 (0.05, 0.16)
		24	3.34 (2.23, 5)	0.89 (0.56, 1.41)	0.27 (0.16, 0.45)
100303601_TGI_at	CCR2	7	2.32 (1.67, 3.22)	0.92 (0.67, 1.26)	0.4 (0.26, 0.6)
		24	1.6 (1.19, 2.14)	0.85 (0.61, 1.18)	0.53 (0.36, 0.79)
Enzymes and signalling molecules in prostaglandin, leukotriene pathways					
100124660_TGI_at	GGT5	7	**4.92 (3.47, 6.98)**	1.25 (0.9, 1.75)	0.25 (0.17, 0.39)
		24	2.64 (1.95, 3.58)	0.91 (0.64, 1.29)	0.34 (0.23, 0.51)
100156386_TGI_at	PTGS1	7	2.11 (1.66, 2.68)	0.64 (0.51, 0.8)	0.3 (0.22, 0.41)
		24	2.04 (1.66, 2.5)	0.54 (0.43, 0.69)	0.27 (0.2, 0.35)
100157709_TGI_at	ALOX15	7	**5.27 (3.29, 8.43)**	0.47 (0.3, 0.74)	0.09 (0.05, 0.18)
		24	**4.57 (3.08, 6.8)**	0.53 (0.33, 0.85)	0.12 (0.06, 0.22)
100159242_TGI_at	PTGS1	7	1.41 (1.19, 1.68)	0.68 (0.58, 0.8)	0.48 (0.38, 0.61)
		24	1.54 (1.33, 1.77)	0.65 (0.55, 0.78)	0.42 (0.34, 0.53)
100309708_TGI_at	PTGS1	7	2.03 (1.6, 2.57)	0.65 (0.52, 0.81)	0.32 (0.23, 0.44)
		24	2.01 (1.65, 2.45)	0.54 (0.42, 0.68)	0.27 (0.2, 0.36)
FcERI and histamine signalling					
100143473_TGI_at	HDC	7	**9.98 (6.15, 16.2)**	0.9 (0.57, 1.43)	0.09 (0.05, 0.18)
		24	2.92 (1.92, 4.46)	0.48 (0.29, 0.78)	0.16 (0.09, 0.31)
100161010_TGI_at	HRH4	7	**6.36 (4.29, 9.43)**	1 (0.69, 1.44)	0.16 (0.09, 0.27)
		24	1.42 (1.01, 1.98)	0.79 (0.53, 1.17)	0.56 (0.34, 0.92)
100161919_TGI_at	HRH4	7	**5.46 (3.51, 8.5)**	1.09 (0.72, 1.66)	0.2 (0.11, 0.35)
		24	1.46 (1, 2.14)	0.7 (0.45, 1.09)	0.48 (0.28, 0.83)
100138809_TGI_at	FCER1A	7	**5.46 (3.36, 8.88)**	0.86 (0.54, 1.36)	0.16 (0.09, 0.28)
		24	**4.06 (2.68, 6.17)**	0.48 (0.3, 0.78)	0.12 (0.07, 0.21)
100153603_TGI_at	FCER2	7	**4.55 (3.55, 5.83)**	1.29 (1.01, 1.63)	0.28 (0.21, 0.39)
		24	1.86 (1.5, 2.31)	0.96 (0.75, 1.23)	0.52 (0.38, 0.7)
Membrane-bound glycoproteins					
100135727_TGI_at	CD1A	7	3.07 (1.83, 5.17)	0.55 (0.34, 0.9)	0.18 (0.09, 0.35)
		24	**7.79 (4.99, 12.15)**	0.63 (0.37, 1.05)	0.08 (0.04, 0.15)
100136515_TGI_at	CD209	7	**4.53 (3.42, 6.01)**	0.97 (0.75, 1.27)	0.21 (0.15, 0.31)
		24	1.89 (1.47, 2.42)	0.85 (0.64, 1.13)	0.45 (0.32, 0.64)
100139590_TGI_at	CD200R1	7	**5.69 (4.19, 7.73)**	1.56 (1.17, 2.09)	0.27 (0.19, 0.4)
		24	1.96 (1.51, 2.55)	0.8 (0.59, 1.09)	0.41 (0.29, 0.59)
100140890_TGI_at	CD200R1	7	3.52 (2.5, 4.95)	1.44 (1.04, 1.98)	0.41 (0.26, 0.65)
		24	1.3 (0.98, 1.74)	0.61 (0.43, 0.86)	0.47 (0.3, 0.73)
100142202_TGI_at	CD1B	7	3.96 (2.45, 6.41)	0.57 (0.36, 0.89)	0.14 (0.07, 0.28)
		24	**10.17 (6.76, 15.31)**	0.68 (0.42, 1.11)	0.07 (0.03, 0.13)
100143809_TGI_at	CD207	7	3.96 (2.46, 6.38)	0.78 (0.5, 1.23)	0.2 (0.11, 0.35)
		24	**4.58 (3.03, 6.91)**	0.64 (0.4, 1.03)	0.14 (0.08, 0.24)
100145467_TGI_at	CD1C	7	3.4 (2.38, 4.86)	0.89 (0.64, 1.25)	0.26 (0.16, 0.42)
		24	**4.82 (3.57, 6.51)**	0.78 (0.55, 1.12)	0.16 (0.1, 0.25)
100161022_TGI_at	CD1C	7	2.96 (1.95, 4.52)	0.68 (0.45, 1)	0.23 (0.13, 0.4)
		24	**5.37 (3.76, 7.66)**	0.59 (0.39, 0.9)	0.11 (0.06, 0.19)
100301303_TGI_at	CD200R1	7	1.34 (1.19, 1.52)	1.03 (0.91, 1.15)	0.76 (0.64, 0.91)
		24	1.05 (0.95, 1.17)	0.86 (0.76, 0.97)	0.81 (0.69, 0.96)
100303713_TGI_at	CD209	7	1.07 (0.99, 1.15)	1.03 (0.96, 1.1)	0.96 (0.87, 1.06)
		24	1.04 (0.97, 1.11)	1.03 (0.95, 1.11)	0.99 (0.9, 1.09)
100311406_TGI_at	CD1C	7	3.01 (1.93, 4.68)	0.65 (0.43, 0.98)	0.22 (0.12, 0.39)
		24	**5.36 (3.69, 7.78)**	0.61 (0.39, 0.95)	0.11 (0.07, 0.2)
Other cytokines, growth factors and their receptors					
100122002_TGI_at	IL32	7	1.7 (1.3, 2.24)	0.67 (0.52, 0.87)	0.39 (0.29, 0.54)
		24	1.41 (1.11, 1.79)	0.53 (0.4, 0.7)	0.38 (0.28, 0.51)
100122958_TGI_at	TNFRSF4	7	3.69 (2.51, 5.41)	0.81 (0.56, 1.18)	0.22 (0.15, 0.33)
		24	2.63 (1.86, 3.71)	0.74 (0.5, 1.09)	0.28 (0.19, 0.42)
100127751_TGI_at	NRG1	7	1.4 (1.23, 1.6)	1.11 (0.99, 1.26)	0.79 (0.66, 0.96)
		24	1.26 (1.13, 1.41)	0.93 (0.81, 1.05)	0.73 (0.61, 0.88)
100130857_TGI_at	TNFRSF10D	7	3.87 (2.74, 5.48)	1.28 (0.92, 1.78)	0.33 (0.21, 0.51)
		24	1.37 (1.01, 1.86)	0.83 (0.59, 1.18)	0.61 (0.4, 0.93)
100134267_TGI_at	CRLF2	7	1.42 (1.1, 1.83)	0.97 (0.76, 1.23)	0.68 (0.49, 0.96)
		24	2.3 (1.86, 2.85)	0.96 (0.75, 1.24)	0.42 (0.3, 0.58)
100136439_TGI_at	FLT3	7	1.14 (0.82, 1.57)	1.29 (0.94, 1.78)	1.14 (0.82, 1.57)
		24	2.59 (1.92, 3.49)	1.3 (0.93, 1.8)	0.5 (0.37, 0.68)
100144830_TGI_at	IL32	7	1.77 (1.38, 2.28)	0.66 (0.52, 0.84)	0.37 (0.28, 0.49)
		24	1.47 (1.18, 1.84)	0.56 (0.43, 0.72)	0.38 (0.29, 0.49)
100147358_TGI_at	IL22	7	**4.86 (2.64, 8.92)**	0.86 (0.48, 1.53)	0.18 (0.09, 0.33)
		24	2.32 (1.34, 4.02)	0.71 (0.39, 1.3)	0.3 (0.17, 0.56)
100148162_TGI_at	IL1RL1	7	3.88 (2.17, 6.91)	1.16 (0.68, 2)	0.3 (0.14, 0.65)
		24	2.04 (1.25, 3.33)	1.06 (0.59, 1.88)	0.52 (0.25, 1.07)
100148210_TGI_at	IL1RL1	7	2.58 (1.74, 3.81)	1.05 (0.72, 1.52)	0.41 (0.25, 0.67)
		24	1.73 (1.24, 2.42)	0.72 (0.49, 1.06)	0.42 (0.26, 0.67)
100150696_TGI_at	NRG1	7	**4.6 (3.08, 6.86)**	1.23 (0.84, 1.79)	0.27 (0.16, 0.44)
		24	**4.37 (3.1, 6.18)**	0.75 (0.51, 1.12)	0.17 (0.11, 0.28)
100153634_TGI_at	GPR171	7	3.01 (2.12, 4.29)	0.92 (0.66, 1.29)	0.3 (0.2, 0.45)
		24	1.98 (1.45, 2.71)	1.06 (0.74, 1.52)	0.54 (0.36, 0.79)
100156398_TGI_at	P2RY6	7	2.79 (2.03, 3.83)	1.08 (0.8, 1.46)	0.39 (0.27, 0.55)
		24	2.29 (1.73, 3.03)	0.79 (0.57, 1.08)	0.34 (0.25, 0.48)
100159528_TGI_at	IL26	7	**4 (2.73, 5.85)**	0.8 (0.56, 1.14)	0.2 (0.12, 0.33)
		24	1.7 (1.23, 2.35)	0.63 (0.43, 0.93)	0.37 (0.23, 0.6)
100300593_TGI_at	NRG1	7	4.1 (2.81, 5.98)	1.24 (0.87, 1.77)	0.3 (0.19, 0.47)
		24	3.88 (2.79, 5.4)	0.71 (0.49, 1.04)	0.18 (0.12, 0.28)
100302151_TGI_at	IL1RL1	7	2.05 (1.4, 3)	1.08 (0.75, 1.55)	0.53 (0.33, 0.85)
		24	1.36 (0.99, 1.89)	1.07 (0.73, 1.57)	0.78 (0.5, 1.24)
100302360_TGI_at	NRG1	7	1.12 (1.01, 1.24)	1.08 (0.98, 1.19)	0.97 (0.84, 1.11)
		24	1.1 (1.01, 1.19)	0.98 (0.89, 1.08)	0.89 (0.78, 1.02)
100302727_TGI_at	NRG1	7	1.13 (1.03, 1.25)	1.06 (0.97, 1.17)	0.94 (0.82, 1.07)
		24	1.04 (0.95, 1.13)	0.94 (0.86, 1.04)	0.91 (0.8, 1.04)
100302783_TGI_at	IL1RL1	7	**8.29 (4.53, 15.16)**	1.44 (0.82, 2.54)	0.17 (0.08, 0.39)
		24	2.75 (1.65, 4.59)	1.09 (0.6, 2)	0.4 (0.18, 0.86)
100302830_TGI_at	TNFRSF4	7	2.81 (2.02, 3.9)	1.09 (0.79, 1.49)	0.39 (0.26, 0.58)
		24	2.05 (1.55, 2.73)	0.83 (0.6, 1.16)	0.41 (0.28, 0.6)
100309572_TGI_at	FLT3	7	1.22 (0.91, 1.64)	0.97 (0.74, 1.27)	0.79 (0.54, 1.17)
		24	2.75 (2.15, 3.51)	1.11 (0.83, 1.5)	0.41 (0.28, 0.59)
100310066_TGI_at	IL32	7	1.78 (1.32, 2.4)	0.61 (0.46, 0.82)	0.35 (0.24, 0.49)
		24	1.51 (1.16, 1.96)	0.47 (0.35, 0.64)	0.31 (0.22, 0.44)
100312593_TGI_at	NRG1	7	1.11 (0.96, 1.27)	0.99 (0.87, 1.13)	0.89 (0.75, 1.06)
		24	0.95 (0.85, 1.07)	0.92 (0.8, 1.06)	0.97 (0.82, 1.14)
100312840_TGI_at	IL1RL1	7	**4.69 (2.56, 8.58)**	1.2 (0.68, 2.12)	0.26 (0.11, 0.57)
		24	2.4 (1.44, 4.02)	1.06 (0.58, 1.93)	0.44 (0.2, 0.95)
Other enzymes not involved in the prostaglandin and leukotriene pathways					
100132327_TGI_at	ADAM19	7	3.09 (2.1, 4.56)	0.91 (0.63, 1.32)	0.3 (0.18, 0.48)
		24	2.43 (1.74, 3.39)	0.65 (0.44, 0.96)	0.27 (0.17, 0.42)
100133255_TGI_at	MMP1	7	**5.02 (3.08, 8.18)**	1.12 (0.7, 1.77)	0.22 (0.12, 0.41)
		24	2.66 (1.75, 4.04)	0.93 (0.57, 1.51)	0.35 (0.19, 0.62)
100141274_TGI_at	MAP2K6	7	**5.37 (3.69, 7.83)**	1.36 (0.95, 1.95)	0.25 (0.16, 0.4)
		24	1.46 (1.05, 2.04)	0.83 (0.57, 1.21)	0.57 (0.37, 0.88)
100145401_TGI_at	MAOA	7	**5.82 (4.1, 8.27)**	1.51 (1.09, 2.11)	0.26 (0.17, 0.4)
		24	2.2 (1.64, 2.97)	1.37 (0.97, 1.94)	0.62 (0.42, 0.93)
100148121_TGI_at	GZMB	7	2.86 (1.84, 4.44)	0.56 (0.37, 0.85)	0.2 (0.11, 0.34)
		24	1.77 (1.22, 2.59)	0.45 (0.29, 0.7)	0.25 (0.15, 0.42)
100300556_TGI_at	MAP2K6	7	**5.38 (3.68, 7.86)**	1.25 (0.87, 1.8)	0.23 (0.15, 0.36)
		24	1.51 (1.08, 2.11)	0.74 (0.51, 1.09)	0.49 (0.33, 0.74)
100301747_TGI_at	ADAM19	7	1.13 (1.01, 1.27)	1.1 (0.99, 1.23)	0.98 (0.85, 1.12)
		24	1.1 (1, 1.22)	0.93 (0.83, 1.04)	0.84 (0.74, 0.96)
100307745_TGI_at	GZMB	7	2.78 (1.85, 4.19)	0.58 (0.4, 0.86)	0.21 (0.13, 0.35)
		24	1.76 (1.24, 2.5)	0.48 (0.32, 0.72)	0.27 (0.17, 0.44)
100309438_TGI_at	MAOA	7	3.25 (2.24, 4.74)	1.49 (1.05, 2.13)	0.46 (0.3, 0.71)
		24	1.67 (1.2, 2.32)	1.03 (0.71, 1.5)	0.62 (0.41, 0.94)
100311427_TGI_at	MAP2K6	7	**6.43 (4.06, 10.18)**	1.6 (1.04, 2.48)	0.25 (0.14, 0.45)
		24	1.15 (0.76, 1.72)	0.69 (0.44, 1.1)	0.61 (0.34, 1.08)
Regulators of the inflammatory responses					
100127993_TGI_at	CISH	7	3.68 (2.84, 4.77)	1.16 (0.91, 1.48)	0.31 (0.23, 0.43)
		24	1.66 (1.33, 2.08)	0.99 (0.76, 1.28)	0.6 (0.44, 0.8)
100149346_TGI_at	SOCS2	7	**4.04 (2.9, 5.61)**	0.91 (0.67, 1.24)	0.23 (0.15, 0.35)
		24	2.09 (1.58, 2.78)	0.89 (0.64, 1.23)	0.42 (0.28, 0.64)
100152491_TGI_at	SOCS1	7	**5.12 (3.82, 6.87)**	1.69 (1.27, 2.24)	0.33 (0.24, 0.46)
		24	1.7 (1.31, 2.2)	1.62 (1.21, 2.17)	0.95 (0.69, 1.31)
100309715_TGI_at	SOCS1	7	1.29 (1.11, 1.49)	1.06 (0.92, 1.21)	0.82 (0.67, 1)
		24	1.16 (1.02, 1.31)	0.98 (0.85, 1.14)	0.85 (0.7, 1.02)
Transcription factors					
100128265_TGI_at	NFATC2	7	2.46 (1.8, 3.36)	0.98 (0.73, 1.32)	0.4 (0.28, 0.56)
		24	1.85 (1.4, 2.44)	0.75 (0.55, 1.03)	0.41 (0.29, 0.56)
100129588_TGI_at	VDR	7	1.84 (1.52, 2.22)	0.99 (0.82, 1.18)	0.54 (0.42, 0.69)
		24	1.23 (1.05, 1.45)	0.9 (0.74, 1.09)	0.73 (0.58, 0.93)
100130323_TGI_at	NFATC2	7	1.09 (0.95, 1.24)	0.96 (0.84, 1.08)	0.88 (0.73, 1.06)
		24	1.12 (1, 1.26)	0.98 (0.86, 1.12)	0.87 (0.73, 1.04)
100137898_TGI_at	IRF4	7	**5.15 (3.29, 8.07)**	1.03 (0.67, 1.57)	0.2 (0.11, 0.35)
		24	3.36 (2.29, 4.93)	0.81 (0.51, 1.27)	0.24 (0.14, 0.41)
100140089_TGI_at	RUNX3	7	3.27 (2.46, 4.36)	1.05 (0.8, 1.37)	0.32 (0.23, 0.45)
		24	2.46 (1.92, 3.14)	0.71 (0.53, 0.95)	0.29 (0.21, 0.4)
100150626_TGI_at	NFATC2	7	2.44 (1.81, 3.29)	0.94 (0.71, 1.25)	0.38 (0.27, 0.55)
		24	1.82 (1.4, 2.37)	0.72 (0.54, 0.98)	0.4 (0.28, 0.56)
100155853_TGI_at	RUNX3	7	3.29 (2.37, 4.56)	1.08 (0.8, 1.48)	0.33 (0.23, 0.47)
		24	2.48 (1.86, 3.31)	0.71 (0.51, 0.98)	0.29 (0.2, 0.4)
100162200_TGI_at	GATA2	7	1.87 (1.58, 2.21)	0.99 (0.85, 1.16)	0.53 (0.41, 0.68)
		24	1.1 (0.95, 1.26)	0.97 (0.82, 1.15)	0.89 (0.7, 1.12)
100163032_TGI_at	VDR	7	3 (2.47, 3.65)	1.11 (0.92, 1.34)	0.37 (0.3, 0.47)
		24	1.74 (1.47, 2.07)	0.88 (0.73, 1.08)	0.51 (0.41, 0.63)
100300539_TGI_at	IRF4	7	**4.28 (2.89, 6.35)**	1.01 (0.7, 1.47)	0.24 (0.15, 0.38)
		24	3.1 (2.21, 4.35)	0.85 (0.57, 1.26)	0.27 (0.17, 0.44)
100301491_TGI_at	GATA2	7	**11.34 (7.04, 18.29)**	0.84 (0.54, 1.33)	0.07 (0.04, 0.14)
		24	2.48 (1.64, 3.75)	0.42 (0.26, 0.68)	0.17 (0.09, 0.31)

A gene is listed in this table if it has at least one significant (two-sided α=0.10) probe set.

a Hours post-allergen challenge.

P=allergen challenge effect: fold change over baseline in placebo group.

F=fold change over baseline in fluticasone group.

Fold change ≥ 4 over baseline in placebo group are indicated in bold.

**Fig. 7:**
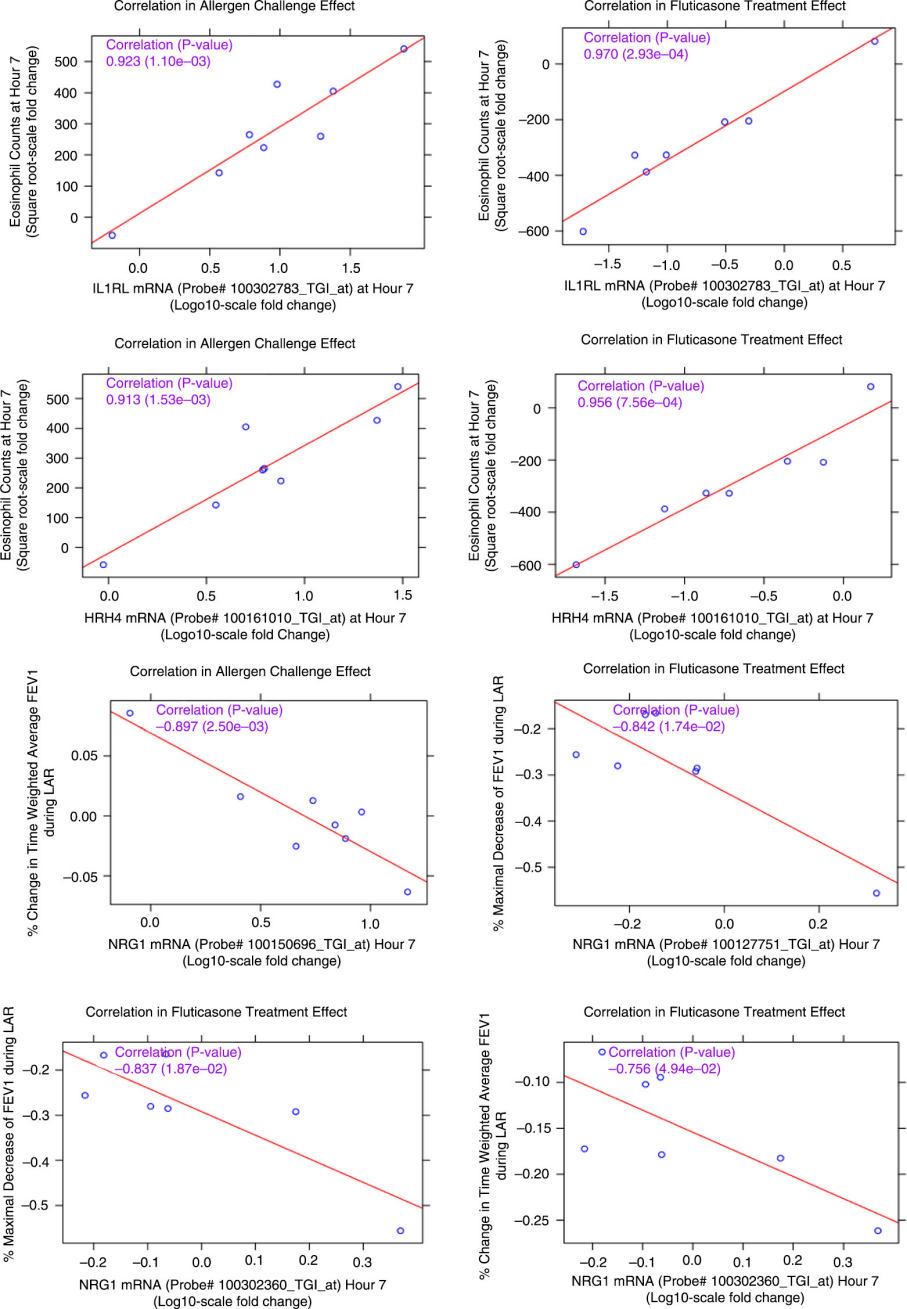
Correlation plots of the most significant probe sets to individual subject clinical measurements (sputum eosinophils and pulmonary lung function tests) for the allergen challenge effect and the FP treatment effect. Correlation coefficients and corresponding *p* values in parenthesis are listed in purple. HRH4: histamine receptor 4; IL1RL1: IL33 receptor; NRG1: neuregulin 1.

The 47 RNA signature set was then used to identify genes correlating with lung function measurements (Table 3) and eosinophil cell counts and percentages (Table 4). Allergen challenge and FP treatment-mediated correlations were independently assessed for each probe set in the signature by estimating correlations at the subject level at 7 and 24 h post-allergen challenge. Correlation plots for the most significant probe sets from each correlation analysis type are represented in Fig. 7. High correlation for some of the probe sets, for example, IL1RL1 and HRH4, and the eosinophil counts from the allergen challenge and the fluticasone treatment effect were observed, with correlation coefficients greater than 0.9 and *p* value between < 0.001 and 0.002. In the ACE analysis, probe sets for NRG1, CCR2, CD1C, MAP2K6, IL26 were negatively correlated with FEV1 measurements at 7 h. In the fluticasone treatment effect, probe sets for NRG1, RUNX3, FLT3 were negatively correlated to the FEV1 measurements at 7 and 24 h. NRG1 was the most significant gene consistently negatively correlated to lung function measurements at 7 h in both the allergen effect and the fluticasone effect analysis with *p* values of and coefficients of correlations in the range of −0.75 (*p* 0.054) to −0.90 (*p* 0.002).

**Table 3:** Correlations between gene expression measurements from the 47 RNA signatures and various FEV1 measurements

Effect of interest	Probe set	Gene symbol	Clinical endpoint	Correlation coefficient (90% confidence interval)	*p*
mRNA at hour 7 and clinical endpoint at hour 7					
Allergen challenge effect	100150696_TGI_at	NRG1	FEV1 Measure II^b^	−0.90 (−0.98, −0.62)	0.002
	100300593_TGI_at	NRG1	FEV1 Measure II^b^	−0.83 (−0.96, −0.44)	0.01
	100124067_TGI_at	CCR2	FEV1 Measure I^a^	−0.79 (−0.95, −0.32)	0.02
	100312593_TGI_at	NRG1	FEV1 Measure I^a^	−0.77 (−0.94, −0.28)	0.025
	100161022_TGI_at	CD1C	FEV1 Measure II^b^	−0.77 (−0.94, −0.27)	0.027
	100303601_TGI_at	CCR2	FEV1 Measure I^a^	−0.76 (−0.94, −0.26)	0.027
	100145467_TGI_at	CD1C	FEV1 Measure II^b^	−0.76 (−0.94, −0.26)	0.027
	100161022_TGI_at	CD1C	FEV1 Measure I^a^	−0.76 (−0.94, −0.25)	0.03
	100147484_TGI_at	CCR2	FEV1 Measure II^b^	−0.75 (−0.94, −0.24)	0.031
	100300556_TGI_at	MAP2K6	FEV1 Measure II^b^	−0.75 (−0.94, −0.24)	0.03
	100311406_TGI_at	CD1C	FEV1 Measure II^b^	−0.74 (−0.93, −0.21)	0.036
	100159528_TGI_at	IL26	FEV1 Measure II^b^	−0.74 (−0.93, −0.21)	0.036
	100303601_TGI_at	CCR2	FEV1 Measure II^b^	−0.74 (−0.93, −0.20)	0.038
Fluticasone treatment effect	100135727_TGI_at	CD1A	FEV1 Measure II^b^	0.87 (0.46, 0.97)	0.012
	100127751_TGI_at	NRG1	FEV1 Measure I^a^	−0.84 (−0.97, −0.39)	0.017
	100302360_TGI_at	NRG1	FEV1 Measure I^a^	−0.84 (−0.97, −0.37)	0.019
	100155853_TGI_at	RUNX3	FEV1 Measure I^a^	−0.77 (−0.95, −0.19)	0.044
	100302360_TGI_at	NRG1	FEV1 Measure II^b^	−0.76 (−0.95, −0.16)	0.049
	100309572_TGI_at	FLT3	FEV1 Measure I^a^	−0.75 (−0.95, −0.16)	0.051
	100133255_TGI_at	MMP1	FEV1 Measure III^c^	−0.74 (−0.91, −0.34)	0.01
mRNA at hour 7 and clinical endpoint at hour 24					
Allergen challenge effect	100148726_TGI_at	CCL17	FEV1 Measure III^c^	−0.80 (−0.95, −0.36)	0.016
	100133255_TGI_at	MMP1	FEV1 Measure III^c^	−0.74 (−0.93, −0.22)	0.035
Fluticasone treatment effect	100145401_TGI_at	MAOA	FEV1 Measure III^c^	0.80 (0.27, 0.96)	0.031
	100301747_TGI_at	ADAM19	FEV1 Measure III^c^	0.80 (0.26, 0.96)	0.031
	100127751_TGI_at	NRG1	FEV1 Measure III^c^	−0.75 (−0.95, −0.14)	0.054
	100155853_TGI_at	RUNX3	FEV1 Measure III^c^	−0.74 (−0.94, −0.12)	0.058
	100309572_TGI_at	FLT3	FEV1 Measure III^c^	−0.74 (−0.94, −0.12)	0.058
mRNA at hour 24 and clinical endpoint at hour 24					
Allergen challenge effect	100157709_TGI_at	ALOX15	FEV1 Measure III^c^	0.77 (0.28, 0.94)	0.025
	100149346_TGI_at	SOCS2	FEV1 Measure III^c^	−0.75 (−0.94, −0.24)	0.031

a % Change in maximal drop of FEV1 during LAR.

b % Change in time weighed average of FEV1 during LAR.

c % Change in FEV1 at hour 24.

FEV1 measure I:% change in maximal drop of FEV1 during LAR; FEV1 measure II:% change in time weighed average of FEV1 during LAR; FEVI measure III:% change in FEV1 at hour 24. Significant probe sets (*p*<0.1 and correlation coefficient > 0.73) are displayed.

**Table 4:** Correlations between the gene expression measurements from the 47 RNA signatures and eosinophils (cell counts and percentages)

Effect of interest	Probe set	Gene symbol	Clinical endpoint	Correlation coefficient (90% confidence interval)	*p*
mRNA at hour 7 and clinical endpoint at hour 7					
Allergen challenge effect	100302783_TGI_at	IL1RL1	Eosinophil counts	0.92 (0.70, 0.98)	0.001
	100161010_TGI_at	HRH4	Eosinophil counts	0.91 (0.67, 0.98)	0.002
	100149346_TGI_at	SOCS2	Eosinophil counts	0.91 (0.66, 0.98)	0.002
	100312840_TGI_at	IL1RL1	Eosinophil counts	0.88 (0.56, 0.97)	0.004
	100301491_TGI_at	GATA2	Eosinophil counts	0.87 (0.53, 0.97)	0.005
Fluticasone treatment effect	100148162_TGI_at	IL1RL1	Eosinophil counts	0.98 (0.92, 1.00)	<0.001
	100312840_TGI_at	IL1RL1	Eosinophil counts	0.98 (0.88, 1.00)	<0.001
	100302783_TGI_at	IL1RL1	Eosinophil counts	0.97 (0.85, 0.99)	<0.001
	100142511_TGI_at	CCL26	Eosinophil percentage	0.97 (0.84, 0.99)	<0.001
	100148726_TGI_at	CCL17	Eosinophil percentage	0.96 (0.80, 0.99)	<0.001
	100161010_TGI_at	HRH4	Eosinophil counts	0.96 (0.79, 0.99)	<0.001
	100134267_TGI_at	CRLF2	Eosinophil percentage	0.93 (0.69, 0.99)	0.002
	100135727_TGI_at	CD1A	Eosinophil percentage	0.92 (0.64, 0.98)	0.004
	100148210_TGI_at	IL1RL1	Eosinophil counts	0.90 (0.59, 0.98)	0.005
	100157709_TGI_at	ALOX15	Eosinophil counts	0.89 (0.52, 0.98)	0.008
	100302151_TGI_at	IL1RL1	Eosinophil counts	0.88 (0.52, 0.98)	0.008
	100162200_TGI_at	GATA2	Eosinophil percentage	0.88 (0.52, 0.98)	0.008
	100125222_TGI_at	IL13	Eosinophil percentage	0.88 (0.51, 0.98)	0.008
	100163032_TGI_at	VDR	Eosinophil counts	0.88 (0.51, 0.98)	0.008
	100302151_TGI_at	IL1RL1	Eosinophil percentage	0.88 (0.50, 0.98)	0.009
	100142202_TGI_at	CD1B	Eosinophil percentage	0.87 (0.48, 0.97)	0.01
	100124660_TGI_at	GGT5	Eosinophil counts	0.87 (0.46, 0.97)	0.011
	100143473_TGI_at	HDC	Eosinophil counts	0.87 (0.46, 0.97)	0.012
mRNA at hour 7 and clinical endpoint at hour 24					
Allergen challenge effect	100135727_TGI_at	CD1A	Eosinophil percentage	0.91 (0.65, 0.98)	0.002
	100132327_TGI_at	ADAM19	Eosinophil percentage	0.87 (0.55, 0.97)	0.004
	100136515_TGI_at	CD209	Eosinophil percentage	0.87 (0.53, 0.97)	0.005
Fluticasone treatment effect	100148210_TGI_at	IL1RL1	Eosinophil counts	0.93 (0.67, 0.99)	0.003
	100309438_TGI_at	MAOA	Eosinophil counts	−0.92 (−0.98, −0.64)	0.003
	100309708_TGI_at	PTGS1	Eosinophil counts	0.92 (0.65, 0.98)	0.003
	100124660_TGI_at	GGT5	Eosinophil counts	0.92 (0.63, 0.98)	0.004
	100156386_TGI_at	PTGS1	Eosinophil counts	0.89 (0.53, 0.98)	0.008
	100302151_TGI_at	IL1RL1	Eosinophil counts	0.88 (0.51, 0.98)	0.009
	100136515_TGI_at	CD209	Eosinophil percentage	0.88 (0.49, 0.97)	0.01

Significant probe sets (*p*<0.1 and correlation coefficients > 0.86) are displayed.

## Discussion

In this study, a RNA signature in sputum induced by the allergen challenge and reversed with fluticasone was identified. A subset of these genes, known to regulate the key inflammatory responses associated with allergic asthma, correlated with clinical endpoints and may constitute potential PD biomarkers of response to fluticasone.

Th2 responses have been traditionally described as playing a central role in the pathophysiology of asthma, although not all patients share a Th2 inflammatory pattern (32). It is striking that in our study the shift toward the Th2 differentiation pathway is a major element of the transcriptional response to the HDM challenge in sputum and is down-regulated following response to fluticasone treatment in the mild asthmatic atopic subjects enrolled in this study. The implications of these results are several-fold.

First, the screening of subjects for dual EAR and LAR responses and the strong homogeneity of our results are consistent with the concept of clustering of clinical asthma phenotypes in which presence of eosinophilic infiltration was identified as one of the key variables (33). Furthermore, clinical phenotypes of asthma have been linked to molecular signatures and pathways in a study where Th2 ‘high’ and ‘low’ phenotypes, characterised by differences in airway responsiveness, eosinophilia and airway remodelling, could be differentiated at the molecular level (34). The observed low variability and high effect size obtained for the gene expression measurements in this study are likely due to the careful selection of a homogeneous allergic, corticosteroid responsive subject population characterised by eosinophilic inflammation in response to an allergen challenge.

Second, our results also suggest that gene expression measurements collected in such an allergen challenge platform could guide the development of novel quantitative assays. For instance, one direct application of this technology could be the quantification of the RNAs that correlate the best with eosinophil numbers as a surrogate to the standard sputum eosinophil cell count assays. Another application of our technology would be the selection of PD biomarkers of response to anti-inflammatory treatment in asthma identified from a set of markers that correlate with clinical endpoints.

The results presented here also raised important questions. We identified from our data set two mRNA levels for IL-22 and IL-26, induced by the allergen challenge and reverted to baseline by fluticasone, which have been associated with the Th17 pathway. IL-22 is preferentially produced by Th17 cells in psoriatic skin and mediates the epithelium hyperplasia induced by IL-23 (35). IL-26 is often co-expressed together with IL-17 and IL-22 by activation of Th17 cells; however, its function remains to be further investigated. Despite the significance of IL-22 and IL-26, we were however unable to detect any up or down-regulation of the mRNA levels for IL-17A and IL-17 F, as well as other genes associated with the Th17, therefore providing more support to the concept of a dominant Th2 response in this study.

Another question is whether the observed signature in sputum is due to (i) changes in cell counts, in particular eosinophil cell counts since this cell type is predominantly increased in sputum following a segmental allergen challenge, (ii) up- or down-regulation of messengers within a given cell type, or (iii) a combination of the above. The only way to address this question is to profile individual cell types isolated from sputum; however, the results from our analysis indicated some changes in gene expression that were correlated with cell-type-specific eosinophil cell counts and some that are not, therefore supporting option (iii). On the one hand, we have identified two genes IL1RL1 and HRH4 that correlate extremely precisely with eosinophil cell counts (correlation coefficients > 0.9, *p*<0.002) and are known to be expressed predominantly in eosinophils, basophils and mast cells. RNAs for both genes therefore appear to be excellent surrogates of eosinophil measurements in sputum. Interestingly, polymorphisms in the HRH4 gene were found to be associated with atopic dermatitis (36), while variants of the IL1RL1 gene have been associated with atopic dermatitis and atopic asthma (37). IL1RL1 is also expressed on innate lymphoid cells which produce type 2 cytokines like IL-5 upon stimulation with IL-33 (38). Given the important role that IL1RL1 has in eosinophil function as a receptor for IL-33, this gene might therefore also represent a promising drug target in inflammatory diseases characterised by a strong eosinophilic component correlating with disease symptoms. Then again, we have identified from this study multiple examples of genes that display similar expression pattern upon allergen challenge and fluticasone treatment and which are known to have very different cell type specificity. In particular, chemokines CCL13 and CCL17 have a dendritic specific expression while CCL26 is epithelial specific; similarly CD1A and CD1B are T-cell specific markers. However, as the expression of those genes is up-regulated by the allergen challenge and down-regulated by fluticasone, this suggests that the identified signature cannot be explained uniquely by variations in eosinophil cell counts or percentages and also reflects major transcriptional changes in a large variety of cell types. An analysis of the transcriptional signatures of isolated sputum cell types in combination with the identification of transcriptional modules of genes co-expressed in asthma as previously described in blood (39) could map the relative contribution of each gene and cell type to the inflammatory response.

Finally, we also identified from our analysis a set of RNAs that uniquely correlates with classical lung function measurements. The majority of our signature genes was strongly correlated with FEV1 and related to the ACE (Table 2), contrasting the eosinophilic related genes for which the majority is related to the fluticasone effect (Table 3). The latter provides further evidence for the observed strong relationship between sputum eosinophils and corticosteroid response (34). At 7 h, chemokines or chemokine receptors (CCL17 and CCR2) and membrane-bound glycoproteins such as CD1C correlate to lung function measurements. NRG1, the gene that most significantly correlated to FEV1 measurements at 7 h, is a member of the neuregulin family, which signals through tyrosine kinases of the ErbB3 family. NRG1 induces the expression of the globlet cell mucins MUC5AC and MUC5B in human airway epithelium (40). Its inhibition may therefore represent a novel therapeutic approach for decreasing mucus hypersecretion in respiratory diseases.

It is known that repeated sputum inductions could lead to a neutrophil, eosinophilic and IL-8 response, possibly due to local changes in osmolarity and subsequent epithelial mast cell activation (41). In our study, sputum induction was performed at 22 h pre-allergen challenge (day 1), and at 7 h (day 2) and 24 h (day 3) post-allergen challenge. However, neutrophils (percentage) and IL-8 concentrations remained unchanged (7). Also, identical sputum induction procedures were applied throughout the study as previously described and validated (1, 42), and differences between study periods in this cross-over designed study were analysed, limiting the effect of repeated sputum induction.

In conclusion, our RNA extraction and profiling protocols allowed sensitive assessments of allergen-induced inflammatory signatures in sputum and precise quantification of drug effects on this response in allergic asthmatics. This approach offers novel possibilities for the development of pharmacodynamic biomarkers in asthma.
